# Foraging Behavior and Success of a Mesopelagic Predator in the Northeast Pacific Ocean: Insights from a Data-Rich Species, the Northern Elephant Seal

**DOI:** 10.1371/journal.pone.0036728

**Published:** 2012-05-15

**Authors:** Patrick W. Robinson, Daniel P. Costa, Daniel E. Crocker, Juan Pablo Gallo-Reynoso, Cory D. Champagne, Melinda A. Fowler, Chandra Goetsch, Kimberly T. Goetz, Jason L. Hassrick, Luis A. Hückstädt, Carey E. Kuhn, Jennifer L. Maresh, Sara M. Maxwell, Birgitte I. McDonald, Sarah H. Peterson, Samantha E. Simmons, Nicole M. Teutschel, Stella Villegas-Amtmann, Ken Yoda

**Affiliations:** 1 Department of Ecology and Evolutionary Biology, University of California Santa Cruz, Santa Cruz, California, United States of America; 2 Department of Biology, Sonoma State University, Rohnert Park, California, United States of America; 3 Centro de Investigación en Alimentación y Desarrollo A.C., Unidad Guaymas, Guaymas, Sonora, México; 4 National Marine Mammal Laboratory, Alaska Fisheries Science Center/National Marine Fisheries Service/National Oceanic and Atmospheric Administration, Seattle, Washington, United States of America; 5 Scripps Institute of Oceanography/University of California San Diego, La Jolla, California, United States of America; 6 Marine Mammal Commission, Bethesda, Maryland, United States of America; 7 Institute of Marine Sciences, University of California Santa Cruz, Santa Cruz, California, United States of America; 8 Graduate School of Environmental Studies, Nagoya University, Furo-cho, Chikusa-ku, Nagoya, Japan; University of California Davis, United States of America

## Abstract

The mesopelagic zone of the northeast Pacific Ocean is an important foraging habitat for many predators, yet few studies have addressed the factors driving basin-scale predator distributions or inter-annual variability in foraging and breeding success. Understanding these processes is critical to reveal how conditions at sea cascade to population-level effects. To begin addressing these challenging questions, we collected diving, tracking, foraging success, and natality data for 297 adult female northern elephant seal migrations from 2004 to 2010. During the longer post-molting migration, individual energy gain rates were significant predictors of pregnancy. At sea, seals focused their foraging effort along a narrow band corresponding to the boundary between the sub-arctic and sub-tropical gyres. In contrast to shallow-diving predators, elephant seals target the gyre-gyre boundary throughout the year rather than follow the southward winter migration of surface features, such as the Transition Zone Chlorophyll Front. We also assessed the impact of added transit costs by studying seals at a colony near the southern extent of the species’ range, 1,150 km to the south. A much larger proportion of seals foraged locally, implying plasticity in foraging strategies and possibly prey type. While these findings are derived from a single species, the results may provide insight to the foraging patterns of many other meso-pelagic predators in the northeast Pacific Ocean.

## Introduction

Marine apex predators are an important, yet highly vulnerable, component of pelagic ecosystems [1], [2], but we lack the information necessary to effectively manage these populations over their extensive ranges. The recent dramatic declines of many predator species, and associated impacts to trophic cascades [3], [4], [5], [6], have motivated research programs to study movements, distributions, and foraging behaviors in relation to habitat features [7], [8]. Identifying physical and biological factors associated with foraging success can inform management strategies; however, the challenges associated with obtaining even basic behavioral data often limit or even prohibit effective study. Indeed, major foraging and breeding sites are still being discovered [9], [10]. Pelagic predators are often elusive, far-ranging, and difficult to handle and these characteristics often translate to small sample sizes and/or short study durations. Thus, longitudinal and/or population-level inferences are particularly challenging, and costly, to obtain. Even as advances in biologging technologies mitigate some of these barriers [11], [12], the study of elusive or depauperate species and their habitats remains problematic.

There are several alternate avenues of research that can bypass at least some of the logistical barriers while still yielding informative results. For example, habitat models utilize animal movement data combined with environmental variables to predict distributions within a study range and allow informed extrapolations for novel regions [13], [14]. Another approach uses tracking data from a variety of species to identify cross-taxa hotspots. For example, the Tagging of Pacific Predators (TOPP) program studied an unprecedented 23 species over 10 years [7], [15], providing nearly contiguous coverage of the entire North Pacific Ocean and identified vast regions of elevated predator diversity. Finally, a single-species approach can be used to give insights into the distributions of other species that fill similar ecological roles, but this requires a large sample size with comprehensive geographic coverage. In this study, we apply the single-species approach to gain a better understanding of the links between foraging habitat, foraging success, and natality of a mesopelagic predator in the northeast Pacific Ocean by analyzing the TOPP northern elephant seal (*Mirounga angustirostris*) dataset: one of the largest single-species marine mammal diving/tracking datasets collected to date.

Adult female elephant seals are ideal research platforms to identify key habitats of mesopelagic predators because they dive continuously [16] to exploit resources throughout the northeast Pacific Ocean during two foraging migrations per year and return to land where instruments can be easily attached/removed and body composition can be measured [17], [18], [19]. The two foraging trips consist of a short post-breeding migration (PB; February to May) and a long post-molting migration (PM; June to January). They also exhibit extremely high philopatry, low adult mortality rates, and low instrument loss rates. Collectively, these factors facilitate acquisition of foraging behavior data (movement and diving) with statistically meaningful sample sizes covering the majority of the northeast Pacific basin. This coverage allows us to bypass the uncertainty of predictions using habitat models to observe basin-scale space-use directly.

We expand on previous studies of this species by 1) exploring foraging behavior metrics and associated inter-annual variability in the context of empirically measured foraging success and natality, 2) conducting a spatial analysis identifying persistent mesopelagic foraging habitats across the northeast Pacific Ocean and 3) discussing how other mesopelagic predators may use and respond to changes in the northeast Pacific Ocean. Together, these analyses allow a glimpse into the physical and biological dynamics of the mesopelagic zone and provide a context for examining the foraging patterns of other pelagic predators.

## Methods

### Ethics Statement

The animal use protocol for this research was reviewed and approved by the University of California at Santa Cruz Institutional Animal Care and Use Committee and followed the guidelines established by the Canadian Council on Animal Care and the ethics committee of the Society of Marine Mammalogy. Research was carried out under National Marine Fisheries Service permits: #786-1463 and #87-143.

**Table 1:** Data Summary.

Season	Year	Total	Complete TDR	Complete Track	Paired Track/TDR	Foraging Success	Natality	Known Age
Post-breeding	2004	7	5	5	4	4	–	4
	2005	19	18	15	15	18	–	18
	2006	21	17	15	15	17	–	19
	SABE2006	10	7	4	4	0	–	0
	2007	20	16	17	16	15	–	18
	2008	23	22	21	21	22	–	22
	2009	19	14	13	13	14	–	13
	2010	24	21	18	17	22	–	21
	ANNU PBTotal	133	113	104	101	112	–	115
Post-molting	2004	25	21	10	9	22	23	19
	2005	25	17	17	12	22	22	22
	SABE2005	10	9	10	9	6	6	0
	2006	24	12	15	8	19	20	21
	2007	21	14	19	14	17	19	18
	2008	20	13	11	10	13	14	15
	2009	8	7	6	6	7	7	5
	2010	21	14	13	11	15	15	16
	ANNU PMTotal	144	98	91	70	115	120	116
Total Deployments	ANNU	277	211	195	171	227	120	231
	SABE	20	16	14	13	6	6	0
	Overall	297	227	209	184	233	126	231

Sample sizes by year, season, tagging location, and dataset. The two tagging locations were Año Nuevo, California (ANNU) and Islas San Benito, Mexico (SABE). Years without a prefix are from ANNU.

**Figure 1:**
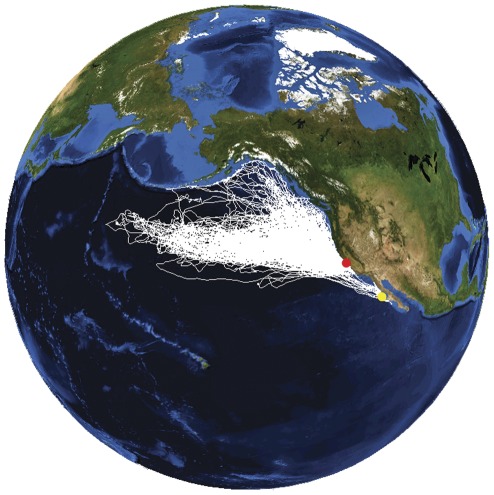
Tracking data from 209 female northern elephant seals from 2004-2010. The map includes 195 tracks from the Año Nuevo, CA, USA colony (red point) and 14 tracks from the Islas San Benito, B.C., Mexico colony (yellow point).

### Field Sites and Animal Handling

Adult female northern elephant seals (*Mirounga angustirostris*) were instrumented at two breeding colonies: Año Nuevo state reserve, California, USA (37° 5′ N, 122° 16′ W; n = 277) and Islas San Benito, Mexico (28° 18′ N, 115° 22′ W; n = 20). The study took place from 2004 to 2010 and included both annual foraging migrations: the short post-breeding migration (PB; February to May) and the long post-molting migration (PM; June to January). We chemically immobilized the seals for instrument attachment and recovery using established protocols [16], [17]. We equipped each seal with a 0.5W ARGOS satellite transmitter (Wildlife Computers, Belleview, WA, USA: SPOT4, SPOT5, MK10-AF; or Sea Mammal Research Unit, St. Andrews, Scotland: SRDL-CTD) using a ∼45 s repetition rate, a time-depth recorder (Wildlife Computers MK9, MK10; or Lotek, St. John’s, NL, Canada: 2310) sampling at least once every 8 s, and a VHF transmitter (MM170B and MM230B, ATS, Isanti, MN, USA).

**Table 2:** Mean (± S.D.) foraging success parameters by year, season, and tagging location (ANNU - Año Nuevo, California and SABE - Islas San Benito, Mexico).

Season	Year	# Females	# Pups	Natality	Mass Gain(kg)	SD	Rate Mass Gain (kg day)	SD	% MassGain	SD	EnergyGain	SD	Rate EnergyGain (MJ/day)	SD
Post-breeding	2004	4	–	–	51.9	21.6	0.6	0.2	17.9	7.6	1047.4	605.3	12.8	7.2
	2005	18	–	–	72.3	23.7	0.9	0.3	22.9	8.7	1105.6	563.0	14.1^1,2^	7.0
	2006	19	–	–	69.0	24.7	0.9	0.3	21.8	8.9	1157.8	566.4	14.5^3,4^	7.5
	2007	18	–	–	82.4	19.1	1.1	0.3	25.8	6.3	1413.6	471.9	19.6	7.5
	2008	22	–	–	74.1	25.1	1.0	0.3	22.4	7.8	1239.0	530.4	16.7	7.3
	2009	13	–	–	87.8	19.9	1.2	0.2	26.8	7.6	1727.3	760.0	23.6^1,3^	9.8
	2010	21	–	–	81.3	19.2	1.1	0.3	23.1	6.2	1645.2	569.9	22.3^2,4^	8.1
	ANNU PB Mean	–	–	–	75.4*	21.6	1.0	0.3	23.1*	7.5	1321.2*	576.9	17.6*	8.0
Post-molting	2004	23	22	95.7	267.0	40.2	1.2	0.2	95.5	14.3	4369.9^1,2^	677.2	19.5	2.9
	2005	22	18	81.8	266.9	65.4	1.2	0.2	98.0	21.7	4146.0	912.4	19.2	3.3
	SABE 2005	6	6	100.0	286.6	36.0	1.3	0.2	120.7	18.2	4108.7	592.7	18.5	3.2
	2006	20	17	85.0	239.6	84.5	1.1	0.3	89.0	30.2	3458.3^1^	1161.3	15.7	4.5
	2007	19	13	68.4	249.7	55.1	1.1	0.2	91.4	21.8	3484.7^2^	846.0	15.2	3.5
	2008	14	12	85.7	260.8	77.6	1.2	0.3	94.1	30.7	3913.2	1323.6	17.5	5.5
	2009	7	6	85.7	271.4	58.0	1.3	0.2	103.2	34.2	4552.2	752.2	21.8	3.5
	2010	15	13	86.7	274.8	52.2	1.2	0.2	94.9	18.0	3630.1	650.6	16.3	2.1
	ANNU PM Mean	–	–	84.1	261.5*	61.9	1.2	0.2	95.2*	24.4	3864.1*	903.3	17.9	3.6

All values are determined from empirical measurement of body composition and mass estimates calculated as the difference between deployment and recovery, after correction for time on land. Identical numeric superscripts denote annual differences within seasons. (*) denotes significant differences across seasons. SABE animals were not included in the statistical comparisons.

**Figure 2:**
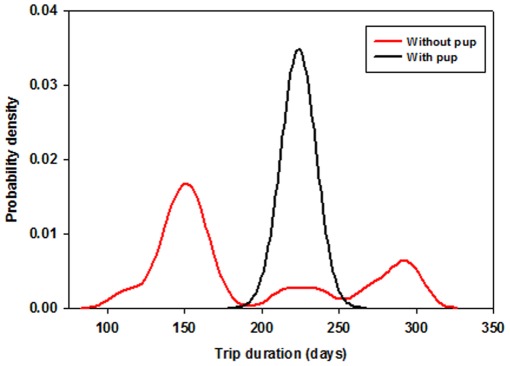
Trip duration for female northern elephant seals observed with (n = 98) and without (n = 17) a pup after the post-molting migration from 2004-2010. Most females that skipped breeding returned outside of the typical breeding season (January – February).

In general, healthy adult female seals were selected at random from the subset of the population carrying flipper tags, allowing us to reference each seal’s age and haulout history [20], [21], [22]. Most seals (78%) were of known age and ranged from 4 to 17 years old. Many of the seals (38%) were instrumented for more than one trip to sea. All analyses and visualizations were corrected to ensure equal representation from each seal when appropriate: mixed models were run with individual as a random effect and kernel densities were down-weighted for repeat deployments. In the 2010 post-breeding season, we intentionally biased animal selection toward an even mix of seals that used coastal and oceanic habitats, based on tracking data from previous deployments, as part of a concurrent study.

**Figure 3:**
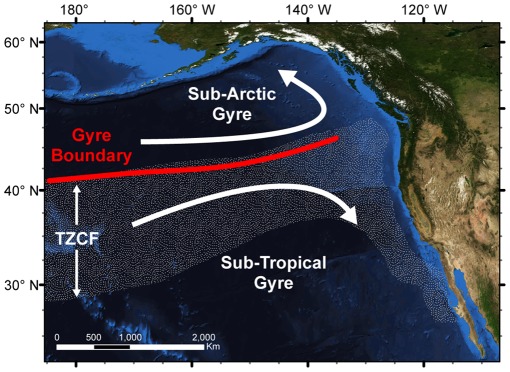
Approximate location of dominant oceanographic features in the northeast Pacific Ocean. The stippled region indicates the annual range of the Transition Zone Chlorophyll Front (TZCF). The location of the gyre-gyre boundary remains stable in contrast to the annual migration of the TZCF.

**Figure 4:**
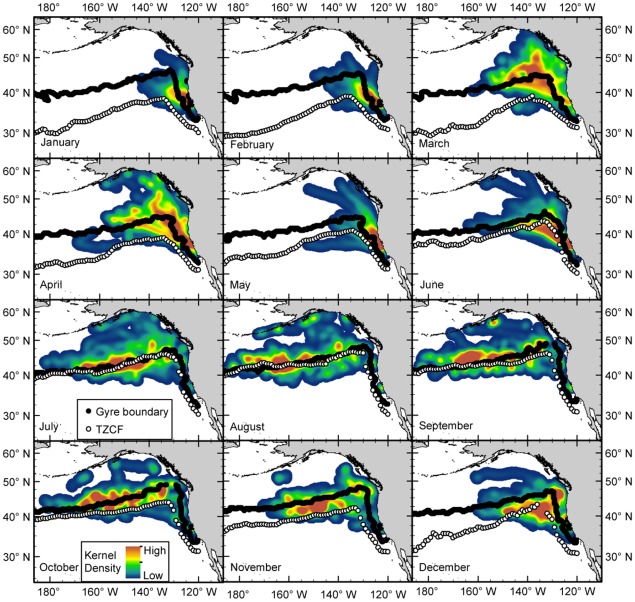
Monthly kernel density distribution of female northern elephant seals from the Año Nuevo, CA colony from 2004-2010. Tracking data were regularized to hourly positions prior to analysis and only complete trips were included (n = 195). The black line shows the monthly position of the gyre-gyre boundary, estimated from the 170 cm absolute dynamic topography climatology contour. White points indicate the position of the Transition Zone Chlorophyll Front, estimated from the 0.2 mg/m^3^ contour. Oceanographic climatologies include data from 2004 through 2008.

### Body Composition

Body composition was measured at both deployment and recovery using the truncated cones technique [23], [24]. Girth and length measurements were taken at 8 locations along the body. Blubber thickness was measured using a handheld ultrasound backfat meter (Scanoprobe, Ithaca, NY) at 18 locations, 3 per girth measurement (except at the head and tail). Mass of the seal at instrument deployment and recovery was measured directly by suspending the seal in a canvas sling from a tripod using a Dyna-Link scale (1,000+/−1 kg). Instruments were attached 6.6 ± 5.4 days prior to departure from the colony and were removed 5.4 ± 4.6 days after return. These lags were of sufficient duration to warrant correction of mass and energy gain estimates. Mass of females at the exact departure and arrival date was estimated from mass measured during deployment (or recovery) using equations derived from serial mass measurements of fasting female seals from previous studies [mass change (kg d^−1^) = 0.51+0.0076 * mass, n = 27, r^2^ = 0.79, p<0.01; [25]]. After arrival from the post-molting migration (i.e. the breeding season), the seals were observed on a daily basis to determine their pup’s birth date. The recovery procedure was always after parturition and the mass of the pup was added to that of the female. Adipose and lean tissue gain was estimated from mass change and body composition, assuming body composition at arrival (or departure) was similar to that during the recovery (or deployment) and that the pup at five days post-partum was 13% adipose tissue [19]. Energy gain was estimated assuming that adipose tissue was 90% lipid, lean tissue was 27% protein with a gross energy content of 37.33 kJ g^−1^ for lipids and 23.5 kJ g^−1^ for protein [19]. These estimates of body composition have been validated against those from dilution of isotopically-labeled water [23].

**Table 3:** Mean (± S.D.) track parameters by year, season, and tagging location (ANNU - Año Nuevo, California and SABE - Islas San Benito, Mexico).

Season	Year	Duration - d	Max Dist – km	Total Dist - km
Post- breeding	2004	83.7 (9.5)	2512.9 (1033.3)	5711.6 (1910.8)
	2005	77.3 (7.9)	2289.4 (511.8)	5059.6 (1013.3)
	2006	76.7 (11.1)	2220.4 (557.6)	5043.9 (1006.9)
	SABE 2006	73.9 (14.6)	1238.0 (1100.6)	2935.3 (1890.6)
	2007	71.2 (8.9)	2086.4 (631.3)	4644.8 (1220.7)
	2008	74.0 (8.9)	2012.9 (358.6)	4813.4 (843.3)
	2009	70.6 (8.2)	2067.5 (544.0)	4778.0 (1029.2)
	2010	73.9 (5.9)	2189.1 (488.2)	5255.5 (789.4)
	ANNU PBMean	74.7 (9.3)*	2140.7 (552.2)*	4913.4 (1068.4)
Post-molting	2004	223.6 (13.5)	3344.9 (840.0)	9355.8 (1251.2)
	2005	214.4 (30.4)	3017.3 (1068.3)	9024.6 (1721.2)
	SABE 2005	210.3 (26.9)	2909.3 (1495.1)	7594.5 (3186.8)
	2006	213.7 (26.5)	3437.5 (964.5)	9775.7 (1261.8)
	2007	223.1 (35.2)	3405.9 (856.3)	10808.0 (2719.8)
	2008	214.2 (30.3)	3267.4 (706.5)	9688.0 (1493.7)
	2009	210.0 (30.9)	2834.3 (1091.6)	10447.9 (2670.8)
	2010	221.9 (27.4)	3079.7 (1128.4)	10099.1 (2144.8)
	ANNU PM Mean	218.5 (25.9)*	3256.9 (944.8)*	9850.0 (1993.1)*

(*) denotes significant differences across seasons. Inter-annual variability was not significant for any parameter. SABE animals were not included in the statistical comparisons.

### Track Data Pre-processing

Raw ARGOS/GPS tracks were truncated according to departure/arrival times identified using the diving record, then processed using a speed/turn-angle filter to remove unlikely position estimates (thresholds: 12 km hr^−1^ and 160°). The filter also examined the secondary position calculations reported by ARGOS and replaced the erroneous primary positions if the speed/angle filter criteria were met. Due to a high prevalence of poor quality ARGOS location classes (predominantly A and B), we used a state-space model to smooth the tracking data and obtain hourly position estimates using the CRAWL package in R [26], [27] that incorporates estimates of at-sea ARGOS error [28].

**Table 4:** Mean (± S.D) diving parameters by year, season, and tagging location (ANNU - Año Nuevo, California and SABE - Islas San Benito, Mexico).

Season	Year	# Dives	% Diving	Depth - m	Duration - min	PDI - min	% Transit	% Foraging	% Drift	% Benthic
Post-breeding	2004	5121.4 (412.6)	89.1 (1.4)	433.1 (79.9)	20.9 (1.5)	2.5 (0.2)	26.4 (4.5)	53.4 (20.9)	6.0 (2.7)	14.1 (16.7)
	2005	4609.1 (675.8)	90.4 (1.3)	499.7 (85.9)	22.0 (2.1)	2.3 (0.3)	26.3 (8.5)	55.5 (14.6)	7.5 (3.4)	10.6 (13.7)
	2006	4650.9 (686.3)	90.9 (1.7)	503.4 (50.2)	21.6 (1.5)	2.2 (0.4)	28.1 (8.5)	58.0 (12.4)	8.1 (3.5)	5.9 (5.5)
	SABE 2006	4370.1 (1387.2)	89.5 (2.9)	477.7 (86.4)	23.0 (5.2)	2.6 (0.4)	15.1 (6.9)	56.6 (28.4)	8.1 (4.1)	20.2 (23.2)
	2007	4473.9 (694.9)	91.3 (1.1)	556.0 (39.0)	21.7 (2.8)	2.0 (0.2)	34.1 (11.3)	53.9 (16.3)	6.6 (2.0)	5.4 (14.4)
	2008	4580.1 (786.3)	90.9 (1.3)	541.6 (40.2)	21.4 (1.8)	2.1 (0.2)	35.0 (11.6)	54.6 (12.4)	7.0 (2.6)	3.4 (1.9)
	2009	4222.9 (804.2)	91.7 (0.9)	540.6 (39.2)	22.4 (2.2)	2.0 (0.2)	34.5 (10.4)	54.6 (10.9)	6.9 (1.8)	4.0 (1.7)
	2010	4356.3 (474.3)	91.2 (0.8)	547.7 (38.5)	22.4 (1.5)	2.2 (0.5)	30.6 (9.2)	58.1 (9.7)	7.2 (2.6)	4.1 (1.8)
	ANNU PB Mean	4513.7 (708.3)	91.0 (1.3)	527.6 (61.6)	21.9 (2.0)	2.2 (0.3)	30.6 (10.4)	56.0 (13.4)	7.2 (2.8)	6.1 (9.8)
Post-molting	2004	12121.2 (1249.6)	91.0 (0.8)	487.4 (44.8)	24.3 (1.7)	2.4 (0.3)	30.5 (9.3)	51.7 (13.7)	11.5 (2.5)	6.3 (5.0)
	2005	11677.4 (1140.8)	90.2 (1.3)	497.4 (54.2)	23.6 (3.1)	2.5 (0.3)	31.5 (11.7)	49.8 (14.1)	11.3 (3.7)	7.4 (9.1)
	SABE 2005	12591.3 (1070.1)	89.0 (2.0)	501.4 (64.9)	21.9 (2.1)	2.6 (0.3)	29.3 (12.2)	53.3 (12.3)	10.1 (3.0)	7.4 (10.2)
	2006	11702.1 (942.9)	90.3 (1.4)	503.6 (27.9)	22.9 (2.8)	2.4 (0.3)	30.7 (10.1)	50.2 (15.2)	14.2 (6.5)	4.9 (3.2)
	2007	11643.1 (2008.4)	90.8 (0.9)	504.7 (35.7)	24.2 (2.9)	2.4 (0.3)	32.4 (5.6)	51.1 (7.5)	11.0 (2.8)	5.5 (4.0)
	2008	11558.2 (810.4)	90.7 (0.9)	512.5 (26.8)	25.2 (1.7)	2.7 (0.4)	27.6 (8.1)	56.6 (9.0)	11.5 (1.6)	4.2 (2.2)
	2009	10662.4 (1278.2)	90.9 (1.0)	525.1 (21.5)	25.7 (2.2)	2.6 (0.5)	26.7 (5.8)	57.6 (7.9)	11.4 (1.2)	4.3 (2.4)
	2010	11246.1 (756.6)	91.5 (0.8)	523.8 (26.8)	26.1 (2.5)	2.7 (0.5)	27.1 (6.9)	56.9 (7.3)	12.2 (3.3)	3.9 (2.6)
	ANNU PM Mean	11622.2 (1281.4)	90.8 (1.0)	503.0 (40.9)	24.5 (2.5)	2.5 (0.4)	30.4 (8.8)	51.8 (11.8)	12.0 (3.6)	5.8 (5.4)
Overall ANNU Mean		–	90.9 (1.2)	516.8 (53.2)	23.1 (2.6)	2.3 (0.4)	30.6 (9.7)	54.0 (12.9)	9.5 (4.0)	5.9 (7.8)
Overall SABE Mean		–	89.3 (2.2)	496.2 (70.7)	22.1 (3.6)	2.6 (0.4)	25.6 (13.7)	53.7 (19.3)	9.1 (3.6)	11.7 (17.1)

‘PDI’ refers to the duration of the post-dive interval. The last 4 columns indicate the proportion of each functional dive type. (*) denotes significant differences across seasons. Inter-annual variation was not significant for any parameter. SABE animals were not included in the statistical comparisons.

### Time-depth Recorder Data Pre-processing

Diving data were collected at sampling intervals between 1 s and 8 s and were sub-sampled to 8 s to facilitate comparison. Three instruments sampled with a 20-second frequency, but were otherwise similar. The raw time-series of depth measurements were analyzed in MatLab using the IKNOS toolbox (Y. Tremblay, unpublished). Dives were retained only if exceeding 32 s in duration and 15 m in depth. All dives were then classified into one of four dive types (each with a putative function) using a forced-choice classification program: active-bottom (pelagic foraging), flat-bottom (benthic foraging), drift (food-processing/rest), or v-shape (transit) [29].

**Figure 5:**
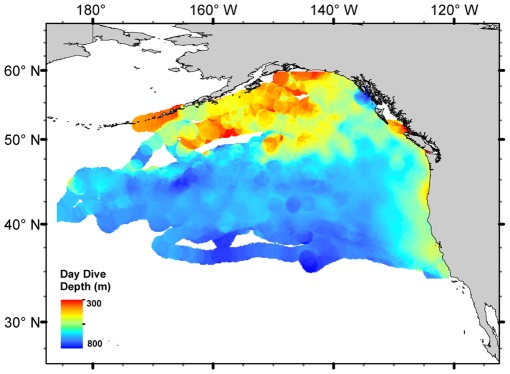
Mean daytime dive depth for northern elephant seals from Año Nuevo, CA seals with a matched and complete diving and tracking record from 2004-2010 (n = 95). Dives are shallower in the northern half of the sub-arctic gyre and coastal regions compared to the transition zone waters.

### Spatial Analyses

To investigate the distribution of individuals throughout the year, we extracted hourly position estimates across all complete tracks by month and generated kernel density plots using a 200 km bandwidth. A weighting (1/# trips) was applied to eliminate the bias associated with repeat deployments on the same individual, as they tend to recapitulate their previous tracks [17]. To explore the relationship between monthly seal distributions and the boundary between the sub-arctic and sub-tropical gyres, we acquired monthly absolute dynamic topography climatologies (AVISO: Topex/Poseidon, ERS-1, ERS-2, Jason-1, and Envisat altimeters) [30] and estimated the boundary as the 170 cm SSH contour.

**Figure 6:**
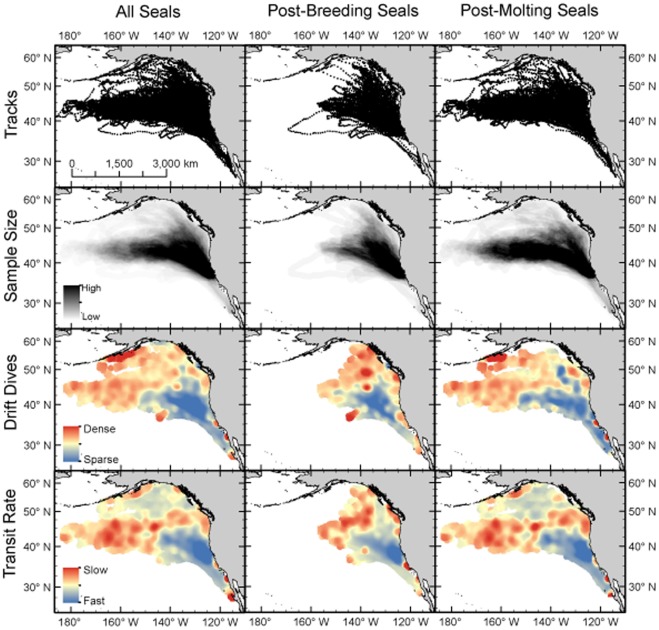
Hotspot analysis (Getis-Ord Gi* statistic) across all years of the study (2004-2010) for female northern elephant seals using two foraging metrics: number of drift dives per day and daily transit rate. Areas in red indicate statistically significant clustering of foraging activity, independent of the number of seals present. Grid cells informed by only one seal were removed to avoid high leverage.

Subsurface thermal structure was explored using temperature data from two seals (one post-molting and one post-breeding) that opportunistically swam directed transects from 40°N to 50°N through the regions of peak inter-annual seal density. Temperature profiles from the ascent (up-cast) of dives were aggregated, smoothed, geo-referenced, and visualized using Ocean Data View (Schlitzer, R., Ocean Data View, http://odv.awi.de, 2011). Temperature profiles (n = 1,186,866) from all seals were processed and contributed to the World Ocean Database as Autonomous Pinniped Bathythermographs (APB; as described by [31]).

**Figure 7:**
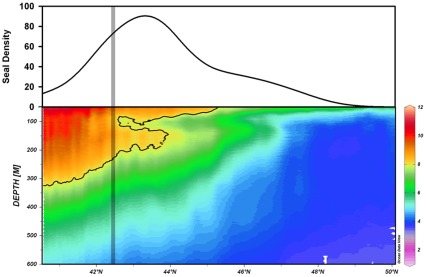
Temperature profile and female northern elephant seal density along a transect of the ∼163W meridian from 40N to 50N. The temperature profile was created from TDR data between 28-July-2005 and 24-August-2005 (seal ID: 2005037; post-molting season). The 8°C isotherm, indicated with a black line, highlights the temperature inversion. The seal density was extracted from the inter-annual August kernel density (see fig. 5). The grey bar shows the position of the gyre-gyre boundary.

To investigate spatial patterns of foraging success across all years of study, we conducted two hotspot analyses for independent verification of trends. The tracking data were first sub-sampled to one position per day, evenly spaced in time. A daily time-scale was selected because many aspects of foraging behavior occur on a diel cycle [17]. Then, two foraging metrics were calculated for each day of the migration: daily transit rate and number of drift dives per day. These metrics have been identified previously from a suite of commonly used diving and movement metrics to be the most indicative of foraging [29].

To identify clustering of elevated foraging activity independent of the number of observations in a particular area, we used the Hotspot Analysis tool (Getis-Ord Gi* statistic) in the Spatial Statistics toolbox of ArcGIS 10. The foraging metric (daily transit rate or number of drift dives per day) was used as the weighting variable. The ‘Zone of Indifference’ setting was used to reduce edge-effects and a radius of 100 km was selected to match the approximate maximum daily displacement of a transiting seal. The points were then converted to a raster using a mean neighborhood analysis on the Z-statistic, again with a radius of 100 km. A mask was applied to remove cells informed by only one seal. Because the two foraging metrics are based on different datasets (surface movements vs. diving behavior), results can be viewed as independent.

### Statistical Analyses

Annual and seasonal effects on behavior and foraging success were analyzed using linear mixed models (SAS 9.2) with individual seal as a random effect subject, and year, season, and their interaction as fixed effects. The subject effect covariance structure was chosen to minimize the model BIC (Bayesian Information Criterion). Fixed effects were evaluated using type III F-tests. When the year by season interaction was significant, post-hoc comparisons were made within seasons by comparing least square means with a Sidak adjustment for multiple comparisons. Model residuals were assessed for approximate normality.

## Results

### Data Summary

Of the 297 foraging migrations, 184 provided a complete dataset (track and dive record) comprising: 25,079 seal-days, 1,267,563 km of horizontal movement, 1,442,695 km of vertical movement, and 1,403,866 dives. Seventy-eight percent of these migrations also had complete foraging success data (pre- and post-deployment morphometric and mass measurements). An additional 70 migrations provided a partial dataset, either a complete TDR record or a complete track, and were included in relevant analyses. Forty-three migrations had only incomplete or missing records and were removed from the analyses. A detailed summary of sample sizes across seasons, years, and locations is provided in Table 1 and a map of spatial coverage in Figure 1.

### Foraging Success and Natality

At the Año Nuevo colony, mass gain during foraging migrations varied with season (F_1,51_ = 866.5, p<0.0001) but not between years (p = 0.52). Overall mean mass gain during the winter post-breeding migration was 75.4 ± 21.6 kg and showed no significant annual variation but wide inter-individual variation. In contrast, annual mass gain during the post-molting migration was 264.6 ± 58.6 kg and varied annually (Table 2). The seasonal differences in mass gain were a function of trip duration as rates of mass gain did not vary between the two foraging trips and showed similar patterns of annual differences within seasons (Table 2). Energy gain calculations, which account for the lean:adipose tissue ratio varied with year (F_6,51_ = 3.4, p<0.01) and season F_1,51_ = 586.7, p<0.0001). Annual differences in absolute energy gain were only present during the post-molting foraging trip and varied by 24% between minimum (2006) and peak (2010) years. Rates of energy gain also varied annually (F_6,51_ = 3.1, p<0.01) but not seasonally (p = 0.62). Annual rates of energy gain varied in post-breeding females (Table 2) but did not result in absolute differences due to compensatory changes in trip duration. Natality rates across years averaged 84% with one strong year in 2004 near 96% and a severe drop in 2007 down to 68%. Of the seals that failed to reproduce, most returned to the colony earlier (n = 11) or later (n = 4) than reproductive seals (Fig. 2). Annual mean rates of energy gain during post-molting foraging trips were not significant predictors of annual natality (p = 0.19). However, individual energy gain rates during this season were significant predictors of pregnancy (Generalized linear mixed model; F_1,20_ = 15.3, p<0.001).

### At-sea Behavior

Seals instrumented at Año Nuevo, CA, USA (ANNU) foraged throughout the northeast Pacific (Figs. 1, 3). With only two exceptions, seals traveled exclusively north of the colony. During the short post-breeding migration, the seals transited to and from the distal point of the track with few periods of intensive search. The seals remained east of the 160°W meridian, likely constrained by time during this short migration. Most seals foraged in the mesopelagic zone but 15% exploited coastal and continental-shelf areas from California to southeast Alaska during at least part of their trip. During the longer post-molting migration, seals foraged in a vast area of the northeast Pacific with nearly complete coverage north of the 40°N parallel and east of the 180° meridian. While a small proportion of seals focused on coastal regions, the Alaska gyre, or seamounts (Maxwell et al, in press), the majority of foraging effort occurred along the northern boundary of the Transition Zone in a dense band from the 180° meridian all the way to the Canadian coast [25], [29] (Fig. 4).

Seals spent an average of 74.7 ± 9.3 days at sea during the post-breeding migration and 218.5 ± 25.9 days during the post-molting migration (Table 3). The seals also traveled farther (+52%) and had longer cumulative paths (+100%) during the post-molting migration. Although no significant inter-annual variability was found, all three tracking metrics were elevated during the 2007 post-molting migration (Table 3).

Overall, seals dived for 91% of their time at sea, with a mean dive duration of 23.1 ± 2.6 min and a maximum of 109 minutes. Mean dive duration and mean post-dive interval were significantly longer during the post-molting migration than the post-breeding migration (F_1,2_ = 26.4, 36.3 respectively, p<0.05). Active-bottom dives made up the greatest percentage of dives (54.0%), followed by V-shape (30.6%), drift dives (9.5%), and flat-bottom dives (5.9%) (Table 4). While tracking and diving data clearly indicate most seals feed in the pelagic zone, 19 out of 211 seals were at least partly benthic feeders (>10% flat-bottom dives) and five were predominantly benthic (>30% flat-bottom dives). The proportion of dive types across seasons remained relatively consistent with two exceptions; the mean proportion of drift dives was higher during the post-molting migration (Table 4, F_1,42_ = 121.5, p<0.0001) and the mean proportion of foraging dives was greater during the post-breeding migration (F_1,42_ = 5.6, p = 0.02).

The overall mean dive depth was 516 ± 53.2 m (maximum 1735 m), but dive depths showed a strong diel pattern resulting in a bimodal distribution. The deep daytime mode was centered at 619 m while the shallow nighttime mode was centered at 456 m. In addition to the diel depth patterns, a diurnal bimodality was also observed for daytime active-bottom dives in 55% of the seals (modes at 385 m and 641 m). Shallow daytime dives were present throughout the range, but occurred most frequently in the northern region of the sub-arctic gyre (Fig. 5).

The highest density of seals occurred in a migratory corridor off the California coast extending northwest to ∼45°N (Fig. 4– April). This pattern reflects the convergence of seals as they leave from, and return to, their home colony with high spatial and temporal fidelity twice per year. Only 5.3% of the migrations ended at a different colony. Monthly density plots show a strong preference for the 40-50°N latitudinal band during both foraging migrations and are strongly associated with the gyre-gyre boundary, identified using absolute dynamic topography climatologies (Fig. 4). The density band also corresponds to the latitude of the Transition Zone Chlorophyll Front (TZCF) [32] during the post-molting migration, but not the post-breeding migration when the TZCF migrates up to 1,000 km south (Fig. 3). The density band is narrow and persistent for the majority of the post-molting migration from July through November and the peak density extends well west of the 160°W meridian. The density estimates during the post-breeding migration were shifted to the east and generally less concentrated, likely a result of the short duration of the post-breeding migration that is spent largely in transit.

Hotspot analysis revealed clusters of intense foraging activity by either slow transit or an elevated rate of drift dives, independent of how many animals visited a particular region (Fig. 6). These maps clearly down-weight the importance of the area close to the colony, predominantly used as a migration corridor, and highlight successful feeding throughout the Transition Zone and waters to the north, including the continental margins. The patterns were reasonably consistent for both behavioral metrics, indicating agreement between independently-derived diving and movement metrics.

### Subsurface Thermal Structure

To explore possible subsurface thermal features that may influence the distribution of prey species and other mesopelagic predators, we generated a temperature profile of the water column by using the data collected by a seal (ID: 2005037) that swam a direct and continuous transect along the ∼163°W meridian from 50°N to 40°N during the middle of the post-molting foraging migration (from late July to late August; Fig. 7). The multi-year density of seals along this transect was extracted from the August density plot (Fig. 4). The temperature profile indicates an inversion layer at ∼100 m depth and the latitudinal range of this inversion layer corresponds with peak seal density (Fig. 7). The peak seal density was slightly north of the gyre-gyre boundary, as identified by the absolute dynamic topography.

### Distance to Foraging Areas

To address the behavioral impacts of added transit time and, by extension, reduced time in prime foraging habitat, we compared seals instrumented at Año Nuevo, California (ANNU) to concurrent deployments at the Islas San Benito, Mexico (SABE) colony 1,150 km to the southeast during the post-molting 2005 migration. While none of the diving or tracking metrics were significantly different (Tables 3, 4), a much higher proportion of SABE seals foraged exclusively within 500 km of their home colony on the continental shelf (i.e. local seals): 20% of the SABE seals and only 4% of the ANNU seals. After excluding these local seals, proportional mass gain (but not absolute mass gain) was higher for the SABE seals (98% vs. 117%, p = 0.057).

## Discussion

We collected a dataset from female northern elephant seals that combines a large sample size, broad geographic extent, and at-sea foraging success metrics with a direct link to reproductive success. This is a unique combination that allows us to (1) describe the at-sea diving and movement behavior of foraging seals in the context of empirically measured foraging success and natality, (2) identify persistent physical features in the environment that correspond to foraging effort, and (3) discuss how other mesopelagic predators may use and respond to changes in the northeast Pacific Ocean.

### Foraging Success and Natality

In elephant seals, and capital breeding systems in general, the energy acquired during a foraging migration helps to determine whether a female will give birth to a pup and provide enough energy during the short lactation period [33], [34]. Because the post-molting migration coincides with fetal development, foraging success during this migration has the potential to directly impact both maternal investment and overall reproductive success. We found that the individual rate of energy gain during the post-molting foraging migration was a strong predictor of natality. This suggests that individual energy reserves can have important impacts on breeding decisions. Previous studies have focused on weaning mass because it is a relatively easy metric to collect and integrates the total maternal investment [35]. In this study, we collected natality data by following individuals with ARGOS satellite tags and determining their reproductive status regardless of when they returned to the colony. Most seals that failed to reproduce returned well outside of the typical breeding season and would have been inadvertently excluded from a traditional survey. Therefore, previous studies may have overestimated natality and reproductive success.

### At-sea Behavior

We collected foraging behavior data at a finer temporal resolution than possible a decade ago, but movement and diving statistics were largely consistent with previous reports [17], [18] (Tables 2, 3, 4). By collecting a much larger sample size, we gained the ability to explore variation in foraging parameters at scales ranging from the individual to the population. Large variability between individuals was detected in diving parameters, which could indicate variability in the quality or distribution of the prey field. For example, individual variability accounted for 33% of the variation in diving depth and 50% of the variation in dive duration, as detected by the random subject effect in the linear mixed model. Diving behavior was relatively consistent between the post-breeding and post-molting migrations, with the exceptions of mean dive duration, mean post-dive interval, and proportions of dive types. Longer dives and surface intervals could indicate a difference in vertical prey distribution, but a concomitant increase in dive depths was not observed. Dive duration increased linearly with trip progression, so the duration of dives was likely driven by an increase in physiological condition attained through continuous diving during the migration. Elephant seals are known to increase oxygen stores and, therefore, diving capacity, during their time at sea [36]. Drift dives, which serve as food-processing dives [37], made up a larger proportion of dives during the post-molting migration and this may simply result from a larger proportion of time in the prime foraging areas and proportionally less time in transit. The majority of seals with more than 10% flat-bottomed dives spent the distal portion of their foraging trip in coastal regions. This validates the delineation of flat-bottom dives as putative benthic dives and also highlights the importance of many coastal areas to female seals. Previous studies have identified a dramatic sexual segregation of foraging strategies in which males forage on the benthos from the California coast to the Aleutian Islands while females forage in pelagic waters [17], [18]. Although this general pattern remains accurate, we observed benthic foraging in a small number of seals across all years of the study, suggesting females rely on both pelagic and benthic resources [38].

The dive depths of most seals showed a clear diel pattern, consistent with targeting vertically migrating prey species. In the northeast Pacific, prey distributions in both the vertical and horizontal dimensions are poorly understood, but acoustic studies identifying deep scattering layers generally show peak density at shallow depths relative to elephant seal foraging dives [39]. Therefore, the elephant seals are exploiting a prey resource that has yet to be adequately characterized. Despite the abundance of sympatric predators, there are few detailed reports of feeding behavior. In the horizontal domain, the seals exhibit shallower dive depths in the northern region of the subarctic gyre, which closely match the distribution of primarily myctophid species collected from net trawls [39]. A more detailed understanding of the dynamics at lower trophic levels, especially at large spatial scales, would be invaluable in explaining the movement decisions of the seals.

The hotspot and kernel density analyses show the importance of the Transition Zone for elephant seals, which is consistent with prior work [25], [29]. The mixing of cold nutrient-rich waters of the sub-polar gyre and warm nutrient-poor waters of the subtropical gyre is thought to be a major driver of productivity and, therefore, an indirect attractor for a variety of higher predators [30], [32]. The Transition Zone Chlorophyll Front (TZCF; 0.2 mg m^-3^) was previously identified as a convenient surface feature to track this boundary and it served as a strong predictor of predator abundance [32]. Female elephant seals show a strong affinity to the TZCF during much of the summer and autumn, but the seals remain in northern waters while the TZCF migrates up to 1,000 km southward during the winter (Fig. 4). In contrast to the dynamic surface layer, the latitude of the actual gyre-gyre boundary (determined using absolute dynamic topography climatologies) remains quite stable across seasons and years [30]. Therefore, the elephant seals appear to utilize the gyre-gyre boundary during both migrations rather than track surface features such as the TZCF, which explains the previously enigmatic northward migration of seals during the winter post-breeding migration. Elephant seal distributions also show an association with subsurface thermal structure. The highest seal density is associated with temperature inversions at depths of 150 to 200 m. Elephant seals feed primarily at depths of 400 to 600 m, extending well below these inversions, but productivity in shallower water may sustain these deeper communities. This is feasible given the large diel vertical migrations of many potential prey species [39]. These finding are comparable with those of southern elephant seals (*M. Leonina*) foraging in deep water at frontal boundaries in the southern ocean [40], [41]. Although these patterns explain the gross movements of most northern elephant seals, a more detailed analysis is necessary to identify the precise features aggregating prey resources at smaller spatial scales.

For a subset of the elephant seals, the hotspot analyses also show the importance of regions farther north in the sub-arctic gyre. Relatively few animals visit this region, but the large sample size of this study facilitated sufficient coverage. When seals did visit this region, their behavior was indicative of feeding (slower transit rates and elevated frequency of drift dives). Because the hotspot analyses indicate foraging intensity independent of seal density, they are likely an indicator of prey availability. Therefore, the foraging behavior hotspot maps (Fig. 6) may be informative as an estimated prey field for other mesopelagic predators. While the behavioral foraging metrics used for these maps have been validated as proxies for feeding success [29], this analysis can be further refined by using behavior-independent measures of foraging success. For example, at-sea changes in the lipid content of a seal can be estimated quite accurately using TDR data [42] (Schick et al, in review).

### Distance Effects

To address the effects that increased transit costs may have on the behavior and foraging success of a mesopelagic predator, we compared the Año Nuevo (ANNU) colony to the Islas San Benito (SABE) colony 1,150 km to the south. We found a mix of strategies in which most SABE seals traveled north to feed in the same areas as those from ANNU while a subset of the population remained local. The same individuals were tracked during the subsequent post-breeding migration and all seals maintained their strategies, but did not travel as far north during this shorter foraging trip. This may partially explain the findings of a previous study that uses isotopic data to suggest SABE seals feed pelagically ∼8° south of ANNU seals [43]. Fidelity to foraging strategies within individuals, but variation across individuals, has been shown in both northern and southern elephant seals [44], [45]. Foraging success in terms of absolute mass gain was similar between the colonies, but SABE seals were smaller at departure and, therefore, gained proportionally more mass. Taken together, these results could indicate that the energetic benefit of feeding at the gyre-gyre boundary is slightly less favorable for seals from a distant colony, especially in the context of a rich local prey resource. While individuals may be impacted, the elephant seal, as a species, appears well-positioned to withstand environmental perturbations by foraging in several distinct ecoregions.

### Significance to other Species

The northern elephant seal is one of many predators foraging in the mesopelagic zone of the north Pacific. Sperm whales (*Physeter macrocephalus*), beaked whales (e.g. *Berardius Bairdii* and *Ziphius cavirostris*), blue sharks (*Prionace glauca*), and salmon sharks (*Lamna ditropis*) all occupy this region [46], [47], yet relatively little is known about their large-scale foraging patterns. The physical forces driving basin-scale prey distributions identified from the elephant seal dataset are likely relevant to these predators as well. In other systems, oceanographic features, such as fronts and eddies, are consistently identified as important aggregation sites for prey [48]. The ability of predators to locate and exploit these regions in both space and time impacts overall foraging success [40], [49], [50], [51] and, in many cases, reproductive success [52], [53], [54]. In addition, major climate events, such as El Niño Southern Oscillation, have the potential to disrupt either the aggregating features or the predator’s ability to locate the feature and have been shown to impact the foraging behavior and weaning mass of northern elephant seals [35], [55]. With the exception of highly mobile species, it is likely that marine predators require a moderate degree of stability in the prey field to forage and reproduce successfully. In this study, we identified the gyre-gyre boundary in the north Pacific as a key feature associated with the interannual distribution of elephant seals and hypothesize this may be an important region for other species that forage at mesopelagic depths.

### Conclusions

In this study, we used one of the largest mesopelagic predator diving and movement datasets to explore at-sea foraging behavior and inter-annual variability in the context of empirically measured foraging success and natality. We identified high-use areas along the latitudinally stable boundary between the sub-arctic and sub-tropical gyres, which explains the bulk of foraging migration trajectories during both annual migrations. We also showed that elephant seals exhibit a variety of foraging strategies at the population level, which may buffer against the impacts of environmental perturbation.

By studying a relatively accessible species over many years, we can better understand the connections between physical dynamics, predator behavior, foraging success, and demographic consequences in the north Pacific mesopelagic ecosystem. A wide variety of predators occupy this region [7], [46] and by identifying high-use areas that are also geographically stable, management of high-seas ecosystems may become more tangible [56], [57], [58], [59].
